# A Narrative Review of In Vivo Studies on the Role of Reactive Oxygen Species in Ovarian Cancer

**DOI:** 10.3390/antiox15050540

**Published:** 2026-04-24

**Authors:** Jeongmin Lee, Seung Geun Yeo, Hye Ok Kim, Jae Min Lee, Manish Kumar Singh, Sung Soo Kim, Tong In Oh, Dong Choon Park

**Affiliations:** 1 Department of Medicine, College of Medicine, Kyung Hee University, Seoul 02447, Republic of Korea; sallyljm@khu.ac.kr (J.L.); or yeo2park@gmail.com (S.G.Y.); hyeokkim@khu.ac.kr (H.O.K.); 2 Clinical Research Institute, Kyung Hee University Medical Center, Seoul 02447, Republic of Korea; jmlee3042@khu.ac.kr; 3 Department of Otorhinolaryngology—Head and Neck Surgery, College of Medicine, Kyung Hee University Medical Center, Kyung Hee University, Seoul 02447, Republic of Korea; 4 Department of Biochemistry and Molecular Biology, College of Medicine, Kyung Hee University, Seoul 02447, Republic of Korea; manishbiochem@gmail.com (M.K.S.); sgskim@khu.ac.kr (S.S.K.); 5 Department of Biomedical Engineering, College of Medicine, Kyung Hee University, Seoul 02447, Republic of Korea; tioh@khu.ac.kr; 6 Department of Obstetrics and Gynecology, St. Vincent’s Hospital, College of Medicine, The Catholic University of Korea, Suwon 16247, Republic of Korea

**Keywords:** ovarian cancer, reactive oxygen species, preclinical in vivo models, ferroptosis, redox signaling

## Abstract

In ovarian cancer, reactive oxygen species (ROS) are both toxic byproducts and mediators of signaling and stress adaptation, such that the same “ROS change” can suppress or promote tumors in vivo. Here, we integratively summarize how ROS modulation reshapes tumor growth, metastasis, and treatment response in ovarian cancer, based on 22 original in vivo-containing studies that were selected from a five-database search of papers published from January 1990 to December 2025. On the antitumor axis, ROS amplification in xenograft models is accompanied by reduced tumor burden and increased markers of cell death, and can operate through diverse death programs beyond apoptosis, including pyroptosis and ferroptosis. ROS-based anticancer effects may vary depending on whether cytoprotective autophagy is co-induced. For example, in models treated with daphnetin, ROS-dependent cell death occurs together with induction of cytoprotective autophagy and the anticancer effect is strengthened when an autophagy inhibitor is added. In a therapeutic context, autophagy may thus function as an adaptive response in tumor cells to partially buffer ROS-induced stress. Conversely, on the pro-tumor axis, ROS can serve as an upstream signal driving inflammatory and metastatic processes. In a peritoneal metastasis model, GPX1 inhibition-induced ROS elevation was linked to increased metastatic burden. In the context of drug resistance, platinum resistance is proposed to be an adaptive state shaped not by the absolute level of ROS alone, but by integrated ROS-sensing and buffering circuits, the DNA damage response (DDR), and NF-κB networks. In vivo, AMPK–ROS axis activation through ACLY inhibition or resetting of drug responsiveness can be connected to tumor suppression and increased sensitivity. Furthermore, ROS modulation is not limited to tumor cell-intrinsic targets: it can also be linked to therapeutic response reprogramming at the tumor microenvironment (TME) level, such as via regulation of acidity/ROS conditions and coupling to macrophage polarization in immunocompetent syngeneic models. Taken together, these lines of in vivo evidence indicate that, in ovarian cancer, ROS should not be interpreted in a binary “increase/decrease” manner, but rather in terms of redox-buffering capacity, the engaged signaling axes (cell death, DDR, metastasis/inflammation), and interactions with TME factors.

## 1. Introduction

Ovarian cancer, which is a leading malignant tumor affecting women worldwide, imposes a substantial disease burden due to high rates of recurrence with heavy tumor burden after treatment. Among the epithelial ovarian cancers (EOC), high-grade serous ovarian carcinoma (HGSOC) is frequently diagnosed at an advanced stage, often with intraperitoneal dissemination and/or metastasis, making improvement of treatment outcomes an ongoing and clinically important challenge [1]. With respect to the origin, it is difficult to attribute HGSOC solely to the ovarian surface epithelium. Rather, accumulating evidence regarding its continuity with fallopian tube lesions and proposed tumor origins has led to a growing view that cancers of the ovary, fallopian tube, and peritoneum should be understood as a single disease spectrum [2]. This concept has been incorporated into staging systems: The revised FIGO 2014 staging integrates ovarian, fallopian tube, and primary peritoneal cancers into a unified classification and provides clearer definitions of stages based on surgical findings [3]. Therapeutically, EOC is primarily managed with surgical cytoreduction and platinum-based chemotherapy. Although targeted therapies such as PARP inhibitors have recently been introduced, recurrence and drug resistance remain persistent clinical limitations [4]. In this context, researchers need to elucidate the molecular mechanisms that govern tumor-cell stress adaptation, evasion of cell death, and resetting of drug responsiveness, and translate these mechanisms into actionable therapeutic vulnerabilities that can be reproduced within the TME.

Reactive oxygen species (ROS) represent a diverse group of highly reactive chemical molecules derived from the partial reduction of molecular oxygen. These molecules are broadly classified into two main categories: free radicals and non-radical species. Free radicals, which are characterized by the presence of one or more unpaired electrons, primarily include the superoxide anion (O_2_•^−^) and the highly reactive hydroxyl radical (•OH). In contrast, non-radical ROS—which lack unpaired electrons but readily participate in redox reactions—are predominantly represented by hydrogen peroxide (H_2_O_2_) and singlet oxygen (^1^O_2_) [5].

ROS are generated through multiple cellular pathways, including electron leakage during mitochondrial respiration, the activity of nicotinamide adenine dinucleotide phosphate (NADPH) oxidases (a family of membrane-bound enzymes dedicated to ROS production), and transition metal-catalyzed processes (such as the Fenton reaction). They function not merely as toxic by-products but also as mediators of signaling and adaptive responses. This dual nature is highlighted in the context of cancer, where ROS can promote cell proliferation/survival signaling and hypoxic adaptation, but oxidative stress beyond a certain threshold can induce cell death and lead to antitumor outcomes [6]. At the molecular level, this context-dependent duality is orchestrated through the intricate crosstalk between ROS and several master signaling pathways, notably phosphoinositide 3-kinase (PI3K)/protein kinase B (AKT), nuclear factor kappa-light-chain-enhancer of activated B cells (NF-κB), and adenosine monophosphate (AMP)-activated protein kinase (AMPK). The PI3K/AKT pathway is a fundamental driver of cell survival, epithelial–mesenchymal transition (EMT)—a process crucial for metastasis and chemoresistance—and platinum resistance in ovarian cancer [7,8,9]. NF-κB serves as a pivotal pro-survival transcription factor that regulates inflammatory and immune responses, while AMPK acts as a central energy sensor that coordinates metabolic adaptation and redox balance. Moderate levels of ROS can sustain PI3K/AKT activation (e.g., by reversibly oxidizing lipid phosphatases such as PTEN), thereby reinforcing these chemoresistant and metabolic phenotypes. Similarly, ROS frequently act as potent secondary messengers that facilitate the degradation of IκB, driving the nuclear translocation of NF-κB to upregulate inflammatory and metastatic programs. Conversely, in response to severe energy depletion or profound oxidative stress, ROS directly and indirectly activate the AMP-activated protein kinase (AMPK) pathway. As a crucial metabolic checkpoint, this axis orchestrates a delicate adaptive balance between cytoprotective autophagy and programmed cell death. Notably, the dynamic interplay between autophagy and lipid-ROS-driven ferroptosis tightly dictates the ultimate therapeutic responsiveness to cisplatin in ovarian cancer [10]. A comprehensive understanding of how these specific redox-sensitive pathways are wired is essential for contextualizing the role of ROS.

The present review summarizes evidence that is primarily derived from in vivo models, showing that ROS modulation alters ovarian cancer growth, metastasis, and treatment responses. For instance, several preclinical studies found that therapeutic elevation of ROS reduces tumor burden, while other studies showed that tumor growth can accelerate when ROS rises due to a collapse of antioxidant defenses [11,12,13]. These findings suggest that the functions of ROS must be interpreted in a context-dependent manner. Key recurring themes in the in vivo evidence can be summarized as follows. First, induction of tumor-cell death via ROS amplification is linked to diverse death modalities beyond apoptosis, including ferroptosis, pyroptosis, and autophagy regulation. Second, pro-tumor ROS can support tumor progression by coupling with metastatic and inflammatory signaling (e.g., NF-κB) and metabolism-reducing power (NADPH) axes in this setting, ROS acts not as a toxic stressor but as a functional signal. Third, in the context of drug resistance, ROS can serve both as a chemotherapeutic stress and as a signal for survival adaptation. Fourth, ROS modulation may reshape therapeutic responses not only through tumor cell-intrinsic targets but also at the level of the TME.

Accordingly, this review: (1) compares and summarizes how ROS generation and redox-buffering axes in ovarian cancer are connected to in vivo tumor phenotypes; (2) examines under what conditions ROS modulation culminates in activation of tumor cell-death programs versus reinforcement of inflammatory/metastatic programs or maintenance of therapeutic resistance; and (3) proposes considerations for designing combination strategies that target redox pathways in specific contexts, rather than oversimplifying ROS as a standalone target.

## 2. Literature Search Strategy

Although numerous studies have investigated the role of ROS in cancer biology, the literature lacks a structured review focusing specifically on in vivo experimental evidence regarding the role of ROS in ovarian cancer. Therefore, one reviewer (J.L.) conducted a systematic search to identify relevant literature.

Five electronic databases—PubMed, SCOPUS, Cochrane Library, EMBASE, and Google Scholar—were searched for studies published between January 1990 and December 2025. The search strategy included using combinations of the keywords “ovarian cancer”, “in vivo”, “reactive oxygen species”, and “ROS”, along with Boolean operators (“AND”, “OR”) to refine results.

The search was limited to English-language publications. The inclusion criteria were:

1. Original research articles;
2. Studies investigating ROS or free radical-related mechanisms in ovarian cancer;
3. Studies that included in vivo experiments, whether alone or in combination with in vitro data.

The exclusion criteria were:

1. Off-topic studies unrelated to ovarian cancer;
2. Studies that did not include in vivo data;
3. Review articles;
4. Studies that did not directly address ROS or redox signaling;
5. Duplicate publications.

**Figure 1:**
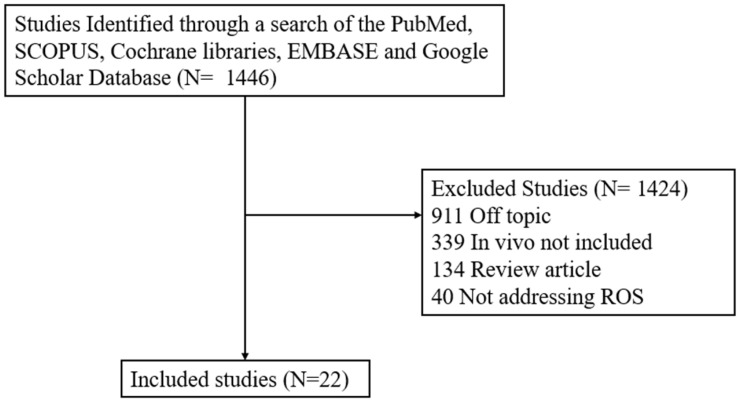
Flowchart of the literature search and selection process for this narrative review.

To ensure that this narrative review was based on a comprehensive and objective overview of the relevant literature, a structured search of the PubMed database was performed. From an initial pool of 1446 studies, 1424 were excluded based on the following predefined criteria: 911 were off-topic, 339 lacked in vivo experiments, 134 were review articles, and 40 did not specifically address ROS mechanisms. Ultimately, 22 primary research articles were included for qualitative analysis and synthesis (Figure 1, Table 1). This selection process represents a transparent workflow that was designed to minimize selection bias while maintaining the thematic focus of a narrative synthesis.

**Table 1:** In Vivo Studies on Reactive Oxygen Species in Ovarian cancer.

Author/ Year/ Reference	Study Design	Sample	Detection Method	Target Substance(s) Associated with ROS	Results/Conclusions
Hu**Y*. *et al., 2005**[11]	Animal (in vivo)—paired subcutaneous SKOV3 xenograft	Female athymic nude mice, *n* = 20	Paired caliper tumor-volume tracking to day 30; terminal tumor weighing	Mn-SOD (SOD2), superoxide (O_2_•^−^), ROS	Mn-SOD-knockdown tumors were ~2× larger than paired controls (*p* = 0.0444). Loss of the mitochondrial antioxidant, Mn-SOD, leads to superoxide accumulation and significantly accelerates ovarian tumor growth in vivo, underscoring the pro-tumorigenic role of ROS elevation following Mn-SOD depletion and highlighting the Mn-SOD/ROS balance as a therapeutic target.
Hseu YC. et al., 2017**[12]	Animal (in vivo)—subcutaneous SKOV-3 xenograft	Female BALB/c-nude mice, 5–6 wk; 3 groups (vehicle, CoQ0 1.5 mg kg^−1^, CoQ0 2.5 mg kg^−1^); i.p. every 4 days for 52 days (*n* = 3 per group)	Caliper tumor-volume tracking, Terminal tumor weighing, H&E histology, TUNEL assay	Coenzyme Q0 (CoQ0), reactive oxygen species (ROS), mitotic index, TUNEL-positive apoptotic cells	Both CoQ0 doses suppressed tumor growth; the high dose decreased the mean volume and weight ≈ 45–50% vs. the vehicle group (*p* < 0.05). Mitotic marker-positive cells fell markedly, while TUNEL-positive apoptotic cells rose in CoQ0 treated tumors (qualitative increase; Figure 11). No bodyweight loss or organ toxicity was observed. CoQ0 curbs the ovarian tumor burden by amplifying ROS-driven apoptosis and reducing proliferation in vivo. The antitumor effect aligns with in vitro data showing that ROS accumulation is indispensable for CoQ0-induced cell death, underscoring that CoQ0-mediated ROS augmentation could be a viable therapeutic avenue against ovarian cancer.
Greenshields A. et al., 2017 [13]	Animal (in vivo)—xenograft	Female C57BL/6 mice; sub-cutaneous ID8 ovarian-tumor model	Terminal tumor weighing (day 50); observation of body-weight/toxicity	Artesunate (endoperoxide ROS generator); ROS; iron (Fe^3+^/holotransferrin); ferroptosis inhibitor (ferrostatin-1)	ART-treated mice developed markedly smaller tumors than controls (mean weight significantly reduced; *p* < 0.05). Systemic ART treatment suppressed ovarian tumor growth in vivo. Mechanistic data (ART cytotoxicity was enhanced by iron loading but blunted by GSH or ferrostatin-1) indicate that the antitumor effect is driven by ROS overproduction and iron-dependent ferroptosis, underscoring ART’s potential to exploit oxidative stress against ovarian cancer.
Wei X. et al.,**2021**[14]	Animal (in vivo)—mouse xenograft	4- to 6-week-old female athymic nude mice (*n* = 5 per group)	Caliper-based longitudinal tumor volumetry, terminal tumor weighing; DCF-based ROS fluorimetry in excised tissue; immunoblotting	ACLY, p-AMPK-α, ROS, PI3K, AKT	shACLY-treated tumors grew markedly slower and were lighter than those of the than NC group (A2780 model; *p* < 0.05 to *p* < 0.0001). In cisplatin-treated A2780/CDDP xenografts, ACLY knockdown further reduced tumor weight vs. that in the NC + cisplatin group (*p* < 0.05). ACLY loss ± cisplatin elevated intratumoral ROS and activated AMPK (*p* < 0.01). ACLY inhibition restores redox stress in vivo, activating the AMPK-ROS axis while dampening PI3K-AKT; this slows tumor growth and re-sensitizes cisplatin-resistant ovarian cancer, underscoring that ACLY/ROS signaling may be exploited as a therapeutic vulnerability.
Gong T-T et al., 2021 [15]	Animal (in vivo)—subcutaneous A2780 xenograft	Female BALB/c-nude mice, 4–5 week old (*n* = 6/group); 5 × 10^6^ A2780 cells flank-injected; groups: vehicle, Iberin 15 mg kg^−1^ (Low) or 30 mg kg^−1^ (High)	Serial caliper tumor-volume tracking, Terminal tumor weighing, RT-qPCR, IHC, Western blot	Reactive oxygen species (ROS); GPX1 (ROS-scavenging enzyme); cleaved caspase-3	Iberin slowed tumor growth from day 8 (Low vs. control *p* < 0.001; High vs. control *p* < 0.0001; High vs. Low *p* < 0.05). The final tumor weight fell by ≈45% (Low) and ≈60% (High) vs. control (*p* < 0.001). GPX1 mRNA and protein levels decreased dose-dependently (Low *p* < 0.001; High *p* < 0.0001). Cleaved caspase-3 was upregulated (*p* < 0.01–0.0001). Iberin suppresses ovarian-tumor growth by elevating intratumoral ROS (as evidenced by downregulation of the antioxidant enzyme, GPX1) and activating ROS-driven apoptosis (↑ cleaved caspase-3). Thus, GPX1 inhibition-mediated ROS enhancement is key to iberin’s in vivo antitumor effect.
Fong MY. et al., 2012 [16]	Animal (in vivo)—subcutaneous A2780 xenograft	5–6-week-old female nude mice (*n* = 5/group); bilateral flank injection of 2 × 10^16^ A2780 cells	Serial caliper tumor-volume tracking; terminal tumor weighing; IHC, TUNEL assay	Withaferin A, reactive oxygen species (superoxide anion), LC3B (autophagy marker), cleaved caspase-3	Dox 1 + WFA 2 mg kg^−1^ combination treatment decreased tumor volume and weight by ~70–80% vs. each mono-treatment (*p* < 0.05–0.01). Low-dose WFA amplified doxorubicin efficacy in vivo by increasing tumor ROS to activate LC3B-mediated autophagy and caspase-3-dependent cell death, achieving marked ovarian tumor suppression with reduced Dox dosage.
Zhang H. et al., 2023 [17]	Animal (in vivo)—subcutaneous CAOV3 xenograft	Female BALB/c nude mice, 5 weeks old; CAOV3 human OC cells (5 × 10^6^); *n* = 4 per arm	Vernier-caliper tumor-volume tracking, terminal tumor weighing, body-weight monitoring	6-Methoxydihydroavicine (6-ME), ROS, p-JNK, p-ERK, mitochondrial fusion proteins MFN1/2 (ROS/MAPK axis)	6-ME shrank the mean tumor volume by ≈50% and weight by ≈45% vs. vehicle on day 14 of post treatment (*p* < 0.05). No bodyweight loss was observed, indicating low systemic toxicity. In vitro mechanistic studies showed that 6-ME raised ROS (per DCF assay), activated JNK/ERK, and suppressed MFN1/2; these effects were reversed by NAC, confirming their ROS dependence. In vivo tumor suppression derives from ROS accumulation leading to JNK/ERK MAPK axis activation and mitochondrial dysfunction; ROS scavenging (NAC) abrogates these effects, underscoring ROS amplification as a driver of 6-ME’s antitumor activity.
Yang X. et al., 2019 [18]	Animal (in vivo)—subcutaneous xenograft mouse model	4-week-old female mice (*n* = 5 per group) injected s.c. with 5 × 10^6^ OVCAR-3 cells; vehicle vs. isoacteoside 30 mg kg^−1^ i.p. thrice weekly for 5 weeks	Serial caliper tumor-volume tracking, Terminal tumor weighing, MTT assay (cell viability), DAPI & Annexin V/PI staining, ROS fluorescent probe, Western blot of AKT/PI3K/mTOR (supporting mechanistic read-outs)	Isoacteoside, ROS, AKT, PI3K, mTOR	Isoacteoside elevated intracellular ROS to ≈ 190% of control levels (*p* < 0.05) in OVCAR-3 cells. In xenografted mice, isoacteoside reduced tumor volume and weight by ~50% vs. control (*p* < 0.05), and ROS-high tumors showed the greatest shrinkage. Isoacteoside’s in vivo antitumor activity is driven by ROS overproduction, which triggers apoptosis and suppresses AKT/PI3K/mTOR signaling to ultimately curb ovarian tumor growth. Thus, ROS amplification is a key therapeutic mechanism in this setting.
Li X. et al., 2024 [19]	Animal (in vivo)—syngeneic intraperitoneal ID8-luc mouse model	6-week-old female C57BL/6 mice; 5 × 10^6^ ID8-luc cells	Weekly IVIS bioluminescence; in vivo DCFH-DA ROS imaging; multiple-IHC for iNOS/CD68; PRKG1 IHC; bulk RNA-seq	Reactive oxygen species (ROS); PRKG1 (cGMP-PKG axis); iNOS & CD68 (M1 macrophage markers)	Combination treatment lowered the tumor luciferin signal vs. that seen with NaHCO_3_ alone (≈50% decrease, *p* < 0.05). Tumor ROS intensity was lower than that seen with Olaparib monotherapy (*p* = 0.0153). The iNOS/CD68 ratio obtained for the combined treatment (1.713) was much greater than those obtained with NaHCO_3_ (*p* = 0.0237) or Olaparib (*p* = 0.0145). RNA-seq revealed 1669 differentially expressed genes (*p* < 0.05) and linked cGMP-PKG pathway downregulation to reduced ROS generation. Neutralization of tumor acidity with NaHCO_3_ amplifies PARP inhibitor efficacy by suppressing the cGMP/PKG pathway to increase ROS scavenging and M1-macrophage infiltration. Thus, ROS depletion in the TME appears to be a key driver of the combination’s antitumor activity.
Yang W. et al., 2020 [20]	Animal (in vivo)—subcutaneous xenograft of TOM40-knock-down vs. control	Female BALB/c nude mice (5 wk); 3 × 10^6^ RMUG-S cells expressing sh-TOM40 or sh-control, *n* = 3–6 per arm	Vernier-caliper tumor-volume curves and terminal weighing, DCFDA, CellROX assays, Luciferase ATP assay	TOM40, intracellular ROS, ATP, AMPK	sh-TOM40 tumors reached only 51% of the control volume at day 68 and weighed significantly less (*p* < 0.001). TOM40 knockdown lowered ATP and elevated ROS, triggering AMPK activation in tumor cells. Xenograft data show that loss of TOM40 increases ROS and depletes ATP, curbing ovarian tumor growth. Thus, targeting the TOM40 → mitochondrial ROS/energy axis represents a potential therapeutic strategy.
Yang C. et al., 2023 [21]	Animal (in vivo)—subcutaneous xenograft mouse model	Female BALB/c mice, 5 weeks old (*n* = 7 per group) implanted with 1 × 10^6^ OVCAR-3 cells	Serial caliper tumor-volume tracking, terminal tumor weighing, immunohistochemistry, Western blot	CBL0137, ROS (indirect via FACT inhibition), BAX, caspase-3, GSDME, SSRP1/SUPT16H	CBL0137 reduced tumor volume and weight by ~50% vs. controls (*p* < 0.001) with no bodyweight loss; tumors showed decreases in the levels of SSRP1/SUPT16H and increases in those of BAX, cleaved caspase-3, and GSDME, which are consistent with ROS-driven pyroptosis. CBL0137-induced FACT inhibition elevates tumor ROS, triggering the ROS → BAX → caspase-3 → GSDME cascade and pyroptotic tumor cell death to suppress ovarian cancer growth in vivo. Targeting this ROS-centered pathway offers therapeutic promise against ovarian cancer.
Fan X. et al., 2021 [22]	Animal (in vivo)—subcutaneous xenograft mouse model	4–6-week-old female BALB/c nude mice (*n* = 5 per group) implanted with 1 × 10^7^ A2780 cells	Serial caliper tumor-volume tracking; terminal tumor weighing; IHC/IF of Ki-67, cleaved caspase-3, LC3, p62	Daphnetin, intracellular ROS, AMPK, Akt, mTOR, autophagy markers (LC3, p62)	Daph alone suppressed tumor volume and weight by ≈ 40–50% vs. control (*p* < 0.05). Adding HCQ further reduced the tumor volume and weight by an additional ≈ 25% vs. Daph alone (*p* < 0.05). Daph elevates tumor cell ROS, driving apoptosis but simultaneously activating cytoprotective autophagy via the AMPK/Akt/mTOR axis. Blocking autophagy with HCQ magnifies the ROS-mediated antitumor effect, underscoring the therapeutic value of combining ROS amplification (here, via Daph) with autophagy inhibition in ovarian cancer.
Zhong Y. et al., 2021 [23]	Animal (in vivo)—subcutaneous xenograft mouse model	Female BALB/c nude mice, 4–6 weeks old, *n* = 5 per group	Serial caliper tumor-volume tracking; terminal tumor weighing; immunohistochemistry for Ki-67, LC3, p62, cleaved caspase-3	Triptolide (TPL), intracellular ROS, JAK2, STAT3, Mcl-1, Beclin1, autophagy markers (LC3, p62)	TPL + DDP reduced tumor volume and weight by ≈50% versus DDP alone (*p* < 0.01); autophagy blockade with CQ partially reversed this effect (*p* < 0.05 vs. TPL + DDP). TPL amplifies cisplatin efficacy in vivo by raising tumor ROS, which suppresses the JAK2/STAT3 pathway, lowers Mcl-1, releases Beclin1, and drives lethal autophagy; inhibiting autophagy or neutralizing ROS blunts this antitumor effect.
Ma J. et al., 2014 [24]	Animal (in vivo)—subcutaneous xenograft of cisplatin-resistant COC1/DDP tumors	6- to 7-week-old BALB/c-nu/nu mice, *n* = 8 per group	Serial caliper tumor-volume curves; slope analysis; terminal tumor weighing; TUNEL staining	Emodin (ROS generator); ROS; MRP1 (ROS-regulated drug-efflux pump)	Emodin + cisplatin slowed growth (slope 2.7 ± 0.4 vs. 3.9 ± 0.6 for cisplatin alone, *p* < 0.05) and increased the apoptotic index to 22.6 ± 3.2 vs. 12.0 ± 2.8 TUNEL-positive nuclei per hpf (*p* < 0.05). Emodin amplifies cisplatin efficacy in vivo by elevating tumor ROS, which suppresses the efflux transporter MRP1 and heightens ROS-mediated apoptosis, markedly enhancing tumor control in platinum-resistant ovarian cancer.
Zhou B. et al., 2019 [25]	Animal (in vivo)—subcutaneous xenograft mouse model	Female BALB/c-nude mice, 4 weeks old (*n* = 6 per group)	Serial caliper tumor-volume tracking, terminal tumor weighing, immunohistochemistry for SOD2, 8-OHdG, γ-H2AX, activated caspase-3	CAM, ROS, antioxidant enzyme SOD2, oxidative-DNA-damage markers 8-OHdG and γ-H2AX	CAM + DDP decreased tumor volume and weight by ≈50% vs. DDP alone (*p* < 0.05 for both). Tumors that received the combined treatment showed markedly lower SOD2 and higher 8-OHdG and γ-H2AX scores on IHC, compared to the single-agent or control groups, indicating that oxidative stress was elevated. Clarithromycin amplifies cisplatin efficacy in vivo by lowering tumor antioxidant capacity (↓ SOD2) and boosting ROS-mediated DNA damage (↑ 8-OHdG, γ-H2AX), resulting in enhanced tumor suppression. Targeting the CAM → ROS increase pathway offers a strategy to overcome cisplatin resistance in ovarian cancer.
Wang H. et al., 2022 [26]	Animal (in vivo)—subcutaneous xenograft mouse model	4- to 6-week-old female BALB/c nude mice (*n* = 5 per group)	Serial caliper tumor-volume tracking; terminal tumor weighing; immunohistochemistry for IDO1 and p53 in excised tumors	IDO1, ROS, p53	Silencing IDO1 slowed tumor growth from day 16 (*p* < 0.05) and combined treatment with cisplatin enhanced this suppression: The final tumor volume and mass fell ≈50–60% vs. the corresponding monotreatments (*p* < 0.01–0.001). IDO1 knockdown boosts ROS accumulation and activates p53-mediated apoptosis, thereby potentiating cisplatin’s antitumor efficacy; targeting the IDO1 → ROS → p53 axis may help overcome platinum resistance in ovarian cancer.
Meng Y. et al.,**2018**[27]	Animal (in vivo)—subcutaneous xenograft efficacy study	Female BALB/c nude mice, 4–6 wk (*n* = 6 per arm) bearing SKOV3-CR tumors	Bi-weekly caliper tumor-volume curves and terminal photography/weight, Immunohistochemistry, TUNEL assay (apoptosis), H&E histology	DUOXA1, ROS, phosphorylated ATR, Chk1	VE-822 alone had little effect, but VE-822 + cisplatin decreased tumor volume by ≈50% vs. cisplatin alone (*p* < 0.01–0.001, two-way ANOVA); *p*ATR staining fell significantly (*p* < 0.05–0.01), and TUNEL-positive cells rose markedly (*p* < 0.001) under the co-treatment. In vivo inhibition of ATR collapses the DUOXA1-driven ROS → ATR-Chk1 survival axis, restoring cisplatin sensitivity and triggering ROS-dependent apoptosis. This suggests that targeting ROS-sustained ATR-Chk1 signaling can overcome platinum resistance in ovarian cancer.
Li Y. et al., 2021 [28]	Animal (in vivo)—subcutaneous xenograft	BALB/c-nude mice, 4–6 wk; 2 × 10^4^ cells µL^−1^ (mixed 1:1 with Matrigel), *n* = 3 per group	Serial caliper tumor-volume tracking, terminal weighing, DCFH-DA flow cytometry, IHC	PSMD4, ROS, NF-κB p65/IκBα, autophagy markers (LC3-II, p62)	shPSMD4 + carboplatin-treated tumors were ≈50% smaller (volume and weight) than those in the NC + carboplatin group (*p* < 0.01). shPSMD4 increased the levels of ROS and IκBα, reduced that of nuclear NF-κB p65 (*p* < 0.05), dampened autophagy (↓ LC3-II/p62), and enhanced apoptosis (↑ cleaved caspase-3). Silencing PSMD4 restores carboplatin responsiveness by amplifying ROS, which suppresses NF-κB signaling, blocks protective autophagy, and drives apoptotic cell death. Targeting the PSMD4 → ROS → NF-κB/autophagy axis may overcome platinum resistance in ovarian cancer.
Yu X. et al., 2024 [29]	In vivo peritoneal-metastasis xenograft	6-week-old female BALB/c nude mice ① metastasis model: *n* = 8 per group ② treatment study: *n* = 5 per group	Longitudinal IVIS bioluminescence imaging and terminal tumor-nodule counting, Flow-cytometric H_2_DCFDA assay for ROS in tumor cells	RUNX1-IT1, STAT1, NuRD–HDAC1, GPX1, ROS, NF-κB	sgRNA-mediated knockdown of RUNX1-IT1 decreased the luminescence flux and metastatic load vs. control (*p* < 0.01; *n* = 8). ASO-RUNX1-IT1 or Entinostat each lowered bioluminescence and nodule counts vs. vehicle (*p* < 0.05); the combination further enhanced these impacts (*p* < 0.01). RUNX1-IT1 overexpression raised intracellular ROS; STAT1 knockdown, HDAC1 knockdown, or NAC (5 mM) treatment normalized ROS and blocked the pro-metastatic effect. In the orthotopic-like mouse model, RUNX1-IT1 accelerates ovarian cancer spread by scaffolding STAT1/NuRD to repress GPX1, causing ROS accumulation and NF-κB activation. Genetic knockdown or pharmacologic inhibition (ASO or an HDAC1 inhibitor) restores the redox balance, curtails ROS-driven signaling, and markedly limits the metastatic burden. These findings suggest that the RUNX1-IT1 → GPX1/ROS axis could be a tractable therapeutic target.
Zhang X. et al., 2021**[30]	Animal (in vivo)—subcutaneous xenograft and tail-vein lung-metastasis mouse models	5-week-old male BALB/c nude mice, 1 × 10^7^ SKOV3 cells stably over-expressing CPT2 (or vector) per mouse; 6 mice/group	Weekly caliper measurement of tumor volume and final tumor weighing, Lung nodule count after 45 days, IHC (Ki-67, TUNEL), DCFH-DA flow cytometry for intratumoral ROS	CPT2, NADPH (FAO-derived), ROS, p-p65 (NF-κB)	CPT2 overexpression slowed tumor growth (≈50% ↓ volume and weight vs. vector, *p* < 0.05) and markedly reduced lung metastatic foci (median ≈1 vs. 7 per mouse, respectively; *p* < 0.05). NADPH rose and ROS levels dropped significantly in CPT2-overexpressing tumors, whereas CPT2 knockdown had the opposite effect (*p* < 0.05). These in vivo data demonstrate that CPT2 acts as a tumor suppressor by enhancing FAO-derived NADPH, thereby lowering ROS and inactivating NF-κB signaling. Loss of CPT2 elevates ROS, driving ovarian cancer growth and metastasis; restoring CPT2 or targeting the ROS/NF-κB axis holds therapeutic promise.

Abbreviation: 6-ME, 6-Methoxydihydroavicine; 8-OHdG, 8-Hydroxy-2′-deoxyguanosine; ACLY, ATP Citrate Lyase; AKT, Protein Kinase B; AMPK, AMP-activated Protein Kinase; ART, Artesunate; ASO, Antisense Oligonucleotide; ATP, Adenosine Triphosphate; ATR, Ataxia Telangiectasia and Rad3-related Protein; BAX, BCL2 Associated X Protein; CAM, Clarithromycin; CDDP/DDP, Cisplatin cis-diamminedichloroplatinum(II); Chk1, Checkpoint Kinase 1; CoQ0, Coenzyme Q0; CPT2, Carnitine Palmitoyltransferase 2; CQ, Chloroquine; DAPI, 4′,6-diamidino-2-phenylindole; DCF/DCFDA/DCFH-DA, 2′,7′-dichlorofluorescein /2′,7′-dichlorodihydrofluorescein diacetate; Dox, Doxorubicin; ERK, Extracellular Signal-Regulated Kinase; FACT, Facilitates Chromatin Transcription; FAO, Fatty Acid Oxidation; GPX1, Glutathione Peroxidase 1; GSDME, Gasdermin E; GSH, Glutathione; H&E, Hematoxylin and Eosin; H2DCFDA, 2′,7′-dichlorodihydrofluorescein diacetate; HCQ, Hydroxychloroquine; HDAC1, Histone Deacetylase 1; hpf, High-power field; IDO1, Indoleamine 2,3-Dioxygenase 1; IF, Immunofluorescence; IHC, Immunohistochemistry; iNOS, Inducible Nitric Oxide Synthase; i.p., Intraperitoneal; IVIS, In Vivo Imaging System; JAK2, Janus Kinase 2; JNK, c-Jun N-terminal Kinase; KD, Knockdown; LC3, Microtubule-associated Protein 1A/1B-light Chain 3; MAPK, Mitogen-Activated Protein Kinase; Mcl-1, Myeloid Cell Leukemia 1; MFN1/2, Mitofusin-1/2; Mn-SOD (SOD2), Manganese-Superoxide Dismutase; MRP1, Multidrug Resistance-Associated Protein 1; mTOR, Mammalian Target of Rapamycin; MTT, 3-(4,5-dimethylthiazol-2-yl)-2,5-diphenyltetrazolium bromide; *n*, number of samples; NAC, N-acetylcysteine; NADPH, Nicotinamide Adenine Dinucleotide Phosphate; NC, Negative Control; NF-κB, Nuclear Factor Kappa B; NuRD, Nucleosome Remodeling and Deacetylase; OC, Ovarian Cancer; *p*, *p*-value; p62, Sequestosome 1 (SQSTM1); PARP, Poly (ADP-ribose) Polymerase; PI, Propidium Iodide; PI3K, Phosphoinositide 3-Kinase; PKG/PRKG1, Protein Kinase G/Protein Kinase cGMP-dependent 1; PSMD4, Proteasome 26S Subunit, Non-ATPase 4; ROS, Reactive Oxygen Species; RT-qPCR, Quantitative Reverse Transcription Polymerase Chain Reaction; RUNX1-IT1, RUNX1 Intronic Transcript 1; s.c., Subcutaneous; SD, Standard Deviation; SEM, Standard Error of the Mean; sgRNA, Single Guide RNA; shRNA, Short Hairpin RNA; SSRP1/SUPT16H, Structure-Specific Recognition Protein 1/Suppressor of Ty 16 Homolog; STAT1/3, Signal Transducer and Activator of Transcription 1/3; TOM40, Translocase of Outer Mitochondrial Membrane 40; TPL, Triptolide; TUNEL, Terminal deoxynucleotidyl transferase dUTP Nick End Labeling; WFA, Withaferin A; γ-H2AX, Phosphorylated Histone H2AX. Symbols: ↑, increase or upregulation; ↓, decrease or downregulation; →, leads to or results in.

## 3. Discussion

### 3.1. In Vivo Evidence That ROS Has Antitumor Functions

#### 3.1.1. Antitumor Effects Driven by ROS Amplification

Globally, ovarian cancer is the eighth-most common cancer in women; it is the most lethal gynecological malignancy, accounting for approximately 4.7% of all cancer deaths. Epithelial ovarian cancer (EOC) represents the vast majority (over 90%) of cases and is characterized by significant clinical and genetic heterogeneity [31]. Carboplatin remains a first-line therapy for patients with EOC, but the emergence of carboplatin resistance significantly reduces treatment benefit and remains a major clinical challenge [32]. Regarding the emergence mechanism of carboplatin resistance proteasome 26S subunit, non-ATPase 4 (PSMD4) functions as a core ubiquitin receptor within the 26S proteasome complex to play vital roles in intracellular protein degradation and quality control. Downregulation of PSMD4 disrupts cellular protein quality control, triggering endoplasmic reticulum stress and a subsequent increase in intracellular ROS. In a subcutaneous xenograft model, combining PSMD4-targeting shRNA with carboplatin yielded an approximately 50% greater reduction in tumor burden compared with monotherapy. In this context, accumulated ROS orchestrates a tumor-suppressive axis by simultaneously attenuating cytoprotective autophagy and suppressing NF-κB signaling to ultimately restore carboplatin sensitivity [33]. Importantly, these results illustrate that the antitumor effects of ROS are highly context-dependent, driving tumor suppression only when ROS elevation is selectively coupled with the disruption of specific survival signaling pathways. Therefore, the potential success of targeting the interplay between redox homeostasis and protein quality control highlights the broader therapeutic potential of exploiting ROS duality in chemoresistant ovarian cancer.

An association between an increase in intracellular ROS and the activation of apoptosis has also been observed with various other anticancer agents, suggesting that there is a conserved mechanism of drug-induced cytotoxicity [34]. Coenzyme Q0 (CoQ0; 2,3-dimethoxy-5-methyl-1,4-benzoquinone), a ubiquinone derivative, possesses potent redox activity and functions as an exogenous ROS generator by undergoing continuous intracellular redox cycling [35]. It can trigger a rapid burst of ROS that exceeds the cellular antioxidant capacity, simultaneously activating apoptotic signaling and inducing compensatory cytoprotective autophagy. In a subcutaneous SKOV-3 xenograft model, CoQ0 significantly suppressed tumor growth. Histological and TUNEL assays indicated a marked increase in apoptosis, supporting the notion that CoQ0-mediated ROS elevation via redox cycling is a primary driver of its antitumor effects [12]. Furthermore, these findings align with the broader theme that intracellular ROS can exert highly context-dependent effects: In this setting, targeted ROS elevation clearly drives antitumor activity.

ROS amplification via metabolic regulation has also been linked to tumor suppression. ATP citrate lyase (ACLY) is a key metabolic enzyme that converts citrate to acetyl-CoA to orchestrate lipid and energy metabolism. The inhibition of ACLY directly induces severe metabolic stress, which consequently leads to AMPK activation and a concurrent increase in intracellular ROS. In a subcutaneous xenograft model utilizing both cisplatin-sensitive (A2780) and cisplatin-resistant (A2780/CDDP) ovarian cancer-derived cells, shRNA-mediated knockdown of ACLY significantly reduced tumor growth and final tumor weight, with elevation of intracellular ROS in the excised tissues. These results support the idea that directed ROS elevation driven by metabolic stress and AMPK activation exerts potent antitumor effects in ovarian cancer models. Specifically, this metabolic-redox rewiring suppresses pro-survival PI3K-AKT signaling to ultimately overcome the acquired cisplatin resistance [14].

ROS accumulation, including its indirect accumulation following inhibition of antioxidant enzymes, has also been associated with tumor suppression following the application of naturally derived compounds. For example, iberin is an isothiocyanate-family compound with antitumor activity. In an A2780 subcutaneous xenograft model, administration of iberin inhibited tumor growth, dose-dependently decreased the mRNA and protein levels of the antioxidant enzyme, glutathione peroxidase-1 (GPX1), and increased the level of cleaved caspase-3. Notably, the ROS scavenger, N-acetylcysteine (NAC), attenuated the iberin-induced antiproliferative and pro-death effects in ovarian cancer cells, suggesting that ROS is functionally involved in iberin-mediated ovarian tumor growth control. Thus, results obtained from both in vitro and in vivo experiments supported the antitumor effects of iberin in ovarian cancer, and the accumulated ROS and reduced GPX1 seen following iberin treatment were found to contribute to inhibiting proliferation and promoting cell death in ovarian cancer cells. In other words, ROS accumulation was linked to increased apoptosis and tumor suppression in this setting [15].

Specific ROS, such as superoxide (O_2_•^−^), have been explicitly implicated in the actions of combination therapy for cancer. Doxorubicin (Dox) is of limited usefulness in cancer treatment due to the risk of severe adverse effects. To minimize Dox-related toxicity, researchers employed a combination strategy with withaferin A (WFA). In an A2780 subcutaneous xenograft model, the WFA + Dox combination reduced tumor volume/weight by ~70–80% compared with the control or monotherapy groups. Across multiple EOC cell lines (A2780, A2780/CP70, and CaOV3), WFA/Dox treatment showed time- and dose-dependent synergistic effects in suppressing proliferation and inducing cell death, suggesting that the required dose of Dox could be lowered. Notably, ROS—particularly O_2_•^−^—was increased relative to controls, and the antitumor effect was amplified accordingly [16].

In ovarian cancer cell-based in vivo models, coupling between ROS elevation and the MAPK axis has also been associated with antitumor effects. Alkaloids extracted from M. cordata (a member of the Papaveraceae family) are known to exhibit anticancer activity in several malignancies. In ovarian cancer, the antitumor efficacy and mechanism of a novel alkaloid from M. cordata fruit, 6-methoxydihydroavicine (6-ME), were explored. Female BALB/c nude mice were maintained under specific pathogen-free (SPF) conditions and injected subcutaneously in the abdomen with CAOV3 cells. Once the tumor volume reached ~100 mm^3^, the mice were randomized into two groups: one group received intraperitoneal 6-ME while controls received vehicle. After 14 days, the mice were sacrificed and tumors were photographed, excised, and weighed. The results revealed that 6-ME reduced tumor volume/weight and increased ROS levels. Further pathway analyses indicated that 6-ME-induced cell death was driven by ROS-mediated activation of mitogen-activated protein kinase (MAPK) signaling and mitochondrial dysfunction in OC cells [17].

In human ovarian cancer cell line-based in vivo models, amplification of total ROS can also translate into antitumor effects accompanied by suppression of growth signaling. In 4-week-old female mice bearing subcutaneous OVCAR-3 xenografts established by injection of 5 × 10^6^ cells, intraperitoneal administration of isoacteoside reduced tumor volume and tumor weight by ~50% compared with controls. In the treated group, intracellular ROS increased to ~190% relative to controls [18].

Conversely, in vivo evidence indicates that antitumor effects can be associated with reduction and/or scavenging of ROS. In an ID8-luc syngeneic intraperitoneal model, combined treatment with NaHCO_3_ and olaparib significantly reduced the tumor signal obtained using an IVIS. Tumor ROS was assessed by in vivo DCFH-DA ROS imaging, which showed that tumor ROS intensity was decreased under the co-treatment compared with olaparib mono-treatment. Thus, this study found that reduction in ROS was associated with antitumor efficacy in an ovarian cancer model [19].

Regulation of mitochondrial transport and function can likewise lead to tumor suppression accompanied by increased ROS. TOM40 is a channel-forming subunit of the mitochondrial translocase complex that is essential for protein import into mitochondria. Among 181 epithelial ovarian cancer cases, TOM40 was overexpressed in 91 (50.3%). TOM40 overexpression showed a significant association with poorer disease-free survival and was also linked to reduced overall survival. In ovarian cancer xenografts, TOM40 knockdown suppressed tumor growth (reduced tumor volume compared with controls) and was reported to be coupled with increased ROS and decreased ATP. In other words, increased intracellular ROS coincided with tumor suppression in this setting [20].

Taken together these reports indicate that, in EOC models, ROS amplification tends to be consistently accompanied by reduced tumor burden along with execution of apoptosis (e.g., TUNEL positivity, increased cleaved caspase-3) and suppression of growth/survival signaling axes (PI3K/AKT/mTOR inhibition, AMPK activation, NF-κB inhibition, etc.). The potential ROS-dependence of the antitumor effects is supported by some studies that found attenuation of antitumor efficacy upon NAC treatment, downregulation of antioxidant enzymes (e.g., GPX1, SOD2), or increased markers of oxidative damage. The latter lines of evidence collectively suggest that ROS elevation may not be merely an epiphenomenon, but rather may be linked to the magnitude of the therapeutic response.

#### 3.1.2. ROS-Mediated Cell Death: Pyroptosis and Lethal Autophagy

In ovarian cancer, ROS amplification seems to be associated not only with apoptosis, but also with the switch toward an alternative death modality, such as pyroptosis. A subcutaneous xenograft tumor model was established using OVCAR-3 cells in 5-week-old female BALB/c mice. The mice were treated with or without Curaxin CBL0137, which was designed to simultaneously regulate p53 and NF-κB and has demonstrated antitumor activity in multiple cancers by suppressing tumor cell proliferation and inducing cell death. After CBL0137 administration, there was no significant difference in bodyweight between groups, but tumor volume/weight was reduced in the treatment group compared with controls. Mechanistic analyses revealed that FACT inhibition-linked ROS elevation was consistent with activation of the BAX–caspase-3–GSDME axis and increases in markers of pyroptosis. In other words, an increase in ROS (or ROS-centered cascade activation) coincided with enhancement of pyroptosis markers in this system. In vivo, CBL0137 also exhibited antitumor effects against ovarian cancer cells. These findings helped define the mechanism and targets through which CBL0137 induces cell death in ovarian cancer cells and suggested CBL0137 as a promising therapeutic candidate for ovarian cancer treatment [21].

There is also in vivo evidence that ROS elevation induces antitumor effects while simultaneously engaging protective adaptive mechanisms, such as autophagy. Daphnetin (Daph) was reported to have antitumor activity. To clarify its antitumor effects and detailed mechanisms in ovarian cancer, a subcutaneous xenograft model was generated by injecting A2780 cells into 4–6-week-old female BALB/c nude mice. Daphnetin treatment reduced tumor volume/weight by ~40–50% compared with controls. Notably, the co-application of hydroxychloroquine (HCQ; an autophagy inhibitor) further reduced the tumor burden by ~25% relative to the results obtained with daphnetin alone, without observable adverse effects. These results suggested that daphnetin triggers ROS-associated tumor cell death via modulation of the AMPK/Akt/mTOR pathway while also inducing cytoprotective autophagy, and that combining daphnetin with an autophagy inhibitor may represent a potential therapeutic strategy for ovarian cancer [22].

In the context of platinum resistance, ROS elevation can couple with lethal autophagy to strengthen antitumor effects. Studies had demonstrated that triptolide (TPL) exerts anticancer activity and enhances sensitivity in cisplatin (DDP)-resistant ovarian cancer by inducing apoptosis in vitro and in vivo. A study on the role and mechanism of TPL-induced autophagy in resistant ovarian cancer examined a cisplatin-resistant SKOV3/DDP subcutaneous xenograft model. In these mice, combination therapy with triptolide (TPL) plus DDP reduced the tumor volume/weight more substantially than DDP alone. The authors interpreted these findings as indicating that TPL increases intracellular ROS and induces lethal autophagy through inhibition of JAK2/STAT3 and Beclin1-related pathways, and the combined antitumor effect was enhanced under conditions accompanied by increased ROS [23].

Taken together, these in vivo studies suggest that ROS amplification in EOC models tends to coincide with reduced tumor burden, execution of apoptosis (e.g., TUNEL positivity, increased cleaved caspase-3), and suppression of growth/survival signaling axes (PI3K/AKT/mTOR inhibition, AMPK activation, NF-κB inhibition, etc.). Moreover, some studies reported attenuation of antitumor efficacy upon NAC treatment, supporting the ROS dependence of these effects. Other studies reported downregulation of antioxidant enzymes (e.g., GPX1 and SOD2) or increases in oxidative damage markers. Collectively, these findings suggest that ROS elevation may not be a mere bystander phenomenon, but could instead be linked to the magnitude of the therapeutic response.

#### 3.1.3. Chemotherapeutic Sensitivity and ROS

Intracellular ROS levels are closely associated with the chemosensitivity of cancer cells. In one study, the authors tested the hypothesis that emodin, a potent ROS generator, can increase the sensitivity of cisplatin (cDDP)-resistant ovarian cancer cells to cDDP cytotoxicity by ROS-dependently suppressing the expression of multidrug resistance-associated protein 1 (MRP1), a major drug efflux transporter responsible for expelling chemotherapeutic agents from the cell [36]. In a platinum-resistant COC1/DDP subcutaneous xenograft model, combined treatment with emodin and cisplatin improved the tumor growth slope and enhanced tumor inhibition compared with cDDP monotreatment, and increased the number of TUNEL-positive cells. Emodin increased ROS generation in COC1/DDP cells, thereby enhancing their sensitivity to cDDP-induced cell death. When emodin and cDDP were administered together, tumor-cell death increased and tumor growth was suppressed in vivo. These results suggested that emodin may function as an adjuvant that enhances the anticancer effects of cDDP via the ROS-associated downregulation of MRP1, indicating that emodin could have potential therapeutic benefit in treating cDDP-refractory ovarian cancer [24].

In vivo evidence was reported from cisplatin-combination settings, which showed changes in ROS-related oxidative DNA damage markers (8-OHdG, γ-H2AX). Cisplatin-based chemotherapy is a first-line treatment for ovarian cancer; however, acquired resistance to cisplatin frequently develops in EOC, creating an urgent need for effective and practical strategies to overcome this problem. Mechanistically, clarithromycin (CAM) was shown to downregulate endogenous antioxidant enzymes and increase ROS levels to augment the cytotoxic effects of cisplatin. In an ovarian cancer xenograft model, the CAM + DDP combination further reduced tumor volume/weight compared with DDP alone. In tumor tissue, decreased SOD2 expression was observed together with increased levels of 8-OHdG and γ-H2AX. These findings suggested that CAM synergizes with cisplatin to inhibit ovarian cancer cell growth and may enhance cisplatin efficacy through a ROS-mediated mechanism [25].

In the platinum-treatment context, ROS accumulation can be linked to modulation of drug sensitivity. Indoleamine 2,3-dioxygenase 1 (IDO1) is a heme-containing dioxygenase that may contribute to chemoresistance in ovarian cancer. In a study on its role in DDP resistance, IDO1 expression levels were measured in tumors from platinum-resistant and -sensitive ovarian cancer patients. In established epithelial ovarian cancer cell-based subcutaneous xenografts, IDO1 silencing (intervention/manipulation) combined with cisplatin treatment (intervention) produced the strongest tumor growth inhibition compared with the control and single-treatment groups (the co-treated final tumor volume/weight was an additional ~50–60% lower than with either monotherapy). The paper proposed that IDO1 inhibition increases ROS accumulation and activates p53-dependent apoptosis, thereby enhancing the antitumor effects of cisplatin, and reported that increased ROS (greater accumulation) relative to controls was associated with increased chemosensitivity [26]. To bridge these preclinical findings with clinical applications, it is essential to consider pharmacological IDO1 inhibitors, such as epacadostat, linrodostat (BMS-986205), and indoximod, which have been extensively evaluated in clinical trials for various solid tumors, including ovarian cancer [37,38]. Mechanistically, IDO1-mediated tryptophan metabolism produces kynurenine-pathway metabolites, which function as key components of the antioxidant and redox-buffering system within the tumor microenvironment. Consequently, the pharmacological blockade of IDO1 not only alleviates immune suppression but also deprives tumor cells of this kynurenine-mediated redox defense, directly exacerbating intracellular ROS accumulation. Furthermore, recent evidence shows that catalytic inhibitors like epacadostat can profoundly influence the non-enzymatic functions of IDO1. By inducing conformational changes in the IDO1 protein, these inhibitors disrupt downstream signaling scaffolds (e.g., the Src/SHP pathway) that intersect with cellular redox-sensing networks [39]. Therefore, the multifaceted ROS-inducing effect of IDO1-targeted therapies provides a strong translational rationale for combining these inhibitors with ROS-generating chemotherapeutic agents to synergistically trigger overwhelming oxidative stress and overcome therapeutic resistance.

Meanwhile, some studies found that chemosensitivity can be increased by blocking the ability of ROS to promote chemoresistance. Acquisition of resistance to platinum drugs is a major obstacle in the clinical use of platinum-based therapy for ovarian cancer. Enhancement of the DNA damage response is a key mechanism contributing to platinum resistance, and researchers addressed how this response is regulated in platinum-resistant ovarian cancer cells. In a SKOV3-CR subcutaneous xenograft model, dual oxidase maturation factor 1 (DUOXA1) was overexpressed in platinum-resistant ovarian cancer cells, leading to excessive ROS production. The elevated ROS levels sustained activation of the ATR–Chk1 pathway, thereby inducing cisplatin resistance in ovarian cancer cells. Accordingly, the authors suggested that inhibiting ROS, DUOXA1, ATR, and/or Chk1 to block this newly identified pathway could effectively overcome cisplatin resistance both in vitro and in vivo [27].

Another study restored platinum sensitivity by blocking a specific ROS-sensing circuit comprising calcium/calmodulin-dependent protein kinase II gamma (CAMK2G)—a versatile kinase that translates calcium and redox signals into cellular responses—and inositol-trisphosphate 3-kinase B (ITPKB), a key enzyme regulating intracellular signaling and cell survival [40]. In ovarian cancer xenograft and PDX (TM0335) models, combination therapy with a CAMK2G inhibitor (KN-93) plus cisplatin markedly reduced tumor volume/weight compared with cisplatin alone. Thus, pharmacologic inhibition of CAMK2G significantly increased ovarian cancer cell sensitivity to cisplatin treatment in vitro and in vivo. Mechanistically, CAMK2G directly senses basal and cisplatin-induced ROS and regulates phosphorylation of ITPKB at serine 174, which directly modulates ITPKB activity and thereby controls cisplatin-induced ROS stress. In this framework, CAMK2G was implicated in regulating redox homeostasis during cisplatin treatment and in the development of cisplatin resistance. The authors interpreted the findings as indicating that a NOX4/ROS-linked circuit maintains resistance, and proposed that blocking the ROS-sensing/preservation circuit collapses resistance and enhances antitumor responses relative to controls [28].

#### 3.1.4. ROS and Iron-Linked Oxidative Damage

An in vivo study of artesunate (ART) demonstrated that ROS plus iron-dependent oxidative stress can lead to antitumor effects. ART is a well-tolerated antimalarial drug that also possesses anticancer activity. In an ID8 ovarian cancer subcutaneous tumor model established in female C57BL/6 mice, the administration of ART (stimulant/stimulus; endoperoxide ROS generator) significantly reduced tumor weight on day 50 post-treatment compared to the control group, and thus successfully inhibited tumor growth. Ovarian cancer cells treated with ART exhibited strongly enhanced ROS and decreased proliferation. ROS-dependent cell cycle arrest occurred at the G2/M phase, whereas ROS-independent cell cycle arrest occurred at the G1 phase and varied depending on the concentration of ART. This suggests that ROS overproduction and iron-dependent oxidative damage could serve as a key axis associated with the antitumor effects of ART.

### 3.2. In Vivo Evidence That ROS Can Promote Tumor Growth and Metastasis

#### 3.2.1. Metastasis Promotion via the ROS–NF-κB Axis

In a peritoneal metastasis model, ROS accumulation can function as a pro-metastatic signal. Dysregulated expression of long-stranded non-coding RNAs (lncRNAs) is closely linked to carcinogenesis. To investigate the mechanisms by which lncRNAs contribute to ovarian cancer pathogenesis, researchers investigated whether the lncRNA, RUNX1-IT1, plays an important role in ovarian cancer progression. In ovarian cancer patient tissues, higher RUNX1-IT1 expression was associated with shorter survival and poorer prognosis. Notably, suppression of RUNX1-IT1 inhibited ovarian cancer cell proliferation, migration, and invasion in vitro, and reduced peritoneal metastasis in vivo. In an in vivo peritoneal-metastasis xenograft model, RUNX1-IT1 was found to suppress GPX1 (an antioxidant enzyme) via a STAT1/NuRD–HDAC1 complex, thereby inducing ROS accumulation. This was accompanied by NF-κB activation and increased metastatic burden. When RUNX1-IT1 was overexpressed, ROS was increased relative to controls; conversely, STAT1 knockdown, HDAC1 knockdown, or NAC (5 mM) treatment normalized ROS levels and blocked the metastasis-promoting effects of RUNX1-IT1 [29].

#### 3.2.2. Collapse of Antioxidant Defense (Especially Mitochondrial ROS Buffering) and Promotion of Tumor Growth

ROS, such as O_2_•^−^ and H_2_O_2_, are continuously generated during metabolic processes in all living organisms. Under normal physiological conditions, intracellular ROS production is balanced by antioxidant enzymes and other redox molecules. The balance between O_2_^−^ generation and removal is critical for maintaining an appropriate cellular redox state. A moderate increase in ROS can promote cellular growth and proliferation, whereas excessive ROS accumulation causes cellular damage such as DNA, protein, and membrane lipid injury. Because of these potentially harmful effects, excess ROS must be rapidly eliminated through diverse antioxidant defense mechanisms, including key enzymes such as superoxide dismutases (SODs), catalase, and various peroxidases. Cytosolic copper/zinc-containing SOD (Cu, Zn-SOD or SOD1) and mitochondrial manganese-containing SOD (Mn-SOD or SOD2) are two essential enzymes that catalyze the conversion of O_2_•^−^ to H_2_O_2_, which is subsequently removed by catalase and peroxidases. Because the mitochondrial respiratory chain is a major intracellular site of O_2_^−^ generation, Mn-SOD plays a crucial role in maintaining the cellular ROS balance [41].

Loss of mitochondrial antioxidant defense can promote tumorigenesis together with accumulation of specific ROS. In a paired subcutaneous SKOV3 xenograft model, Mn-SOD (SOD2)-knockdown tumors grew approximately two-fold larger than paired controls, and Mn-SOD deficiency was reported to induce O_2_•^−^ accumulation (i.e., increased ROS). Thus, increased superoxide/ROS relative to controls was associated with promotion of tumor growth [11].

Metabolism-based redox regulation may also be linked to tumor promotion or suppression. Carnitine palmitoyltransferase 2 (CPT2) is a rate-limiting enzyme involved in regulating fatty acid β-oxidation (FAO). Dysregulated lipid metabolism has increasingly been recognized as being closely related to tumorigenesis. To explore the mechanisms by which CPT2 contributes to ovarian cancer progression, researchers generated SKOV3-based subcutaneous xenograft and tail-vein lung metastasis models. In these mice, CPT2 overexpression inhibited tumor growth and lung metastasis and reduced intratumoral ROS. Conversely, CPT2 downregulation increased ROS and activated NF-κB (p-p65) signaling. In patients, CPT2 expression was reduced in primary ovarian serous carcinomas and showed a significant association with poor survival in ovarian cancer patients. CPT2 was reported to suppress ovarian cancer cell growth and metastasis by inhibiting the G1/S cell-cycle transition and epithelial–mesenchymal transition (EMT), and by inducing cell death. Mechanistically, CPT2 was proposed to increase NADPH generated via fatty acid oxidation, thereby suppressing the ROS/NF-κB signaling pathway and contributing to its antitumor function in ovarian cancer cells [30] (Table 2).

**Table 2:** In vivo evidence for ovarian cancer progression-promoting versus progression-suppressing ROS-related mechanisms.

Direction of Effect on Ovarian Cancer Progression	ROS-Related Factor and Underlying Mechanism
Progression	DUOXA1 overexpression → ROS↑ → ATR–Chk1 pathway
	RUNX1-IT1 → GPX1↓ → ROS↑ → NF-κB↑
	CPT2 downregulation → NADPH↓ → ROS↑ → NF-κB(p-p65)↑
	CAMK2G–pITPKB(Ser174) redox-buffer (ROS-sensing/NOX4-ROS stress alleviation)
	IDO1 → ROS/p53 apoptosis↓ (platinum resistance support)
	CEBPG → SLC7A11↑ (avoidance of ferroptosis, lipid ROS protection)
	Mn-SOD(SOD2) knockdown → superoxide/ROS↑ → tumor growth↑
	TOM40 (mitochondrial import/energy axis)
Suppression	6-ME → ROS↑ → JNK/ERK(MAPK) → apoptosis
	CBL0137 → ROS↑ → BAX–caspase-3–GSDME (pyroptosis)
	Triptolide → ROS↑ → lethal autophagy (±JAK2/STAT3↓)
	PSMD4 downregulation → ROS↑ + autophagy↓
	ACLY↓ → AMPK–ROS pathway↑ → ROS↑
	NaHCO_3_ + olaparib → ROS scavenging + M1 macrophage↑
	CPT2 overexpression → NADPH↑ → ROS↓ → NF-κB↓
	Emodin + cisplatin → ROS↑ → MRP1↓ → apoptosis↑ (platinum-resistant xenograft sensitization)
	Isoacteoside → ROS↑ → tumor suppression
	Clarithromycin + cisplatin → antioxidant capacity↓ (SOD2↓) + oxidative DNA damage↑ (8-OHdG, γ-H2AX)
	Artesunate → ROS overproduction → tumor suppression
	Coenzyme Q0(CoQ0) → ROS-mediated apoptosis (±cytoprotective autophagy) → tumor suppression
	Iberin → GPX1↓ → ROS↑ → apoptosis → tumor suppression
	Daphnetin → ROS↑ → apoptosis + cytoprotective autophagy (AMPK/Akt/mTOR)
	Withaferin A + doxorubicin → (superoxide/ROS↑) autophagy + caspase-3 dependent cell death
	TOM40 knockdown (intervention) → ROS↑ + ATP↓ + AMPK↑

Abbreviation: 6-ME, 6-Methoxydihydroavicine; 8-OHdG, 8-Hydroxy-2′-deoxyguanosine; ACLY, ATP Citrate Lyase; AKT, Protein Kinase B; AMPK, AMP-activated Protein Kinase; ATP, Adenosine Triphosphate; ATR, Ataxia Telangiectasia and Rad3-related Protein; BAX, BCL2 Associated X Protein; CAMK2G, Calcium/Calmodulin-Dependent Protein Kinase II Gamma; CBL0137, Curaxin 0137; CEBPG, CCAAT Enhancer Binding Protein Gamma; Chk1, Checkpoint Kinase 1; CoQ0, Coenzyme Q0; CPT2, Carnitine Palmitoyltransferase 2; DUOXA1, Dual Oxidase Maturation Factor 1; ERK, Extracellular Signal-Regulated Kinase; GPX1, Glutathione Peroxidase 1; GSDME, Gasdermin E; IDO1, Indoleamine 2,3-Dioxygenase 1; ITPKB/pITPKB, Inositol-Trisphosphate 3-Kinase B/Phosphorylated ITPKB; JAK2, Janus Kinase 2; JNK, c-Jun N-terminal Kinase; M1, Macrophage Type 1 (Classically activated macrophage); MAPK, Mitogen-Activated Protein Kinase; Mn-SOD (SOD2), Manganese-Superoxide Dismutase; MRP1, Multidrug Resistance-Associated Protein 1; mTOR, Mammalian Target of Rapamycin; NADPH, Nicotinamide Adenine Dinucleotide Phosphate; NaHCO3, Sodium Bicarbonate; NF-κB, Nuclear Factor Kappa B; NOX4, NADPH Oxidase 4; p53, Tumor Protein p53; p-p65, Phosphorylated p65 (RelA); PSMD4, Proteasome 26S Subunit, Non-ATPase 4; ROS, Reactive Oxygen Species; RUNX1-IT1, RUNX1 Intronic Transcript 1; Ser174, Serine 174; SLC7A11, Solute Carrier Family 7 Member 11; STAT3, Signal Transducer and Activator of Transcription 3; TOM40, Translocase of Outer Mitochondrial Membrane 40; γ-H2AX, Phosphorylated Histone H2AX. Symbols: ↑, increase or upregulation; ↓, decrease or downregulation; →, leads to or results in.

To reconcile the seemingly contradictory outcomes of ROS elevation—where it drives tumor suppression in some contexts (e.g., via lethal autophagy or apoptosis) but promotes tumor progression in others (e.g., via Mn-SOD knockdown)—it is crucial to recognize that ROS function is not a simple binary variable of elevation or reduction [42,43]. Instead, the biological outcome is governed by precise spatiotemporal and chemical factors. First, the specific types of ROS dictate the downstream effects: while H_2_O_2_ is relatively stable and frequently acts as a secondary messenger to activate pro-survival pathways (such as NF-κB or PI3K/AKT), O_2_•^−^ and •OH are highly reactive and prone to inducing irreversible macromolecular damage. Second, subcellular localization is paramount. For instance, mitochondrial ROS accumulation is tightly coupled with mitochondrial dysfunction, membrane depolarization, and intrinsic apoptosis, whereas cytosolic ROS elevation may engage distinct metabolic or inflammatory cascades. Third, the magnitude and duration of the ROS burst are decisive. A moderate, chronic increase in ROS typically preconditions cells to upregulate antioxidant defenses and supports proliferation and metastasis, whereas an acute, overwhelming burst of ROS that exceeds the cellular redox-buffering capacity triggers severe oxidative stress, leading to apoptosis, pyroptosis, or ferroptosis. Ultimately, the divergent phenotypes observed across various ovarian cancer models represent the intersection of these multidimensional factors with the tumor’s specific redox landscape.

### 3.3. Methods to Measure ROS in Preclinical Ovarian Cancer Models

ROS constitute a chemically diverse set of oxidants with distinct reactivities, half-lives, and compartmental preferences. Accordingly, no single assay suffices for all biological contexts [44,45]. Below, we outline commonly used approaches in ovarian cancer models—including several applied by the in vivo studies summarized in this review—highlighting comparability, key differences, and caveats. We then offer pragmatic reporting recommendations. General oxidant indicators, such as DCFH-DA, are widely used to index a composite “oxidative capacity.” Following intracellular de-esterification, DCFH is oxidized to fluorescent DCF via peroxidase- and heme-dependent reactions. While highly practical for both in vitro and in vivo whole-tumor imaging (e.g., in bicarbonate–olaparib models [19], DCF signals lack species specificity and are susceptible to auto-oxidation, photo-oxidation, and artifactual amplification by transition metals. Consequently, DCF readouts should be interpreted broadly as redox imbalance rather than the accumulation of a discrete ROS.

To achieve higher spatial and chemical resolution, compartment-directed and species-targeted probes are often employed. Superoxide-directed probes like dihydroethidium and its mitochondrial analogue MitoSOX Red are particularly useful for mapping mitochondrial ROS changes linked to apoptosis or metabolic perturbations [20]. However, standard fluorescence cannot fully distinguish the specific 2-hydroxyethidium adduct from nonspecific oxidation products, occasionally requiring chromatographic (HPLC/LC-MS) confirmation. For H_2_O_2_, genetically encoded sensors (e.g., HyPer family members and roGFP variants) afford ratiometric, subcellularly targeted live imaging, offering a robust alternative to dye-based methods, despite the prerequisite of stable cellular expression. Furthermore, in contexts where iron-dependent cell death is implicated, lipid peroxidation sensors such as C11-BODIPY are critical for directly aligning ROS measurements with ferroptotic pathways [13,46].

Beyond fluorescent probes, measuring oxidative-damage footprints provides orthogonal, integrative evidence of oxidative stress impacting macromolecules in vivo. Quantifying DNA oxidation (e.g., 8-OHdG or γ-H2AX) or lipid/protein adducts (e.g., 4-HNE, carbonyls) serves as a reliable biological anchor. While these endpoints provide definitive proof of oxidative damage, they integrate stress over time and cannot identify the initiating ROS. Conversely, while electron paramagnetic resonance (EPR) spin trapping remains the chemical gold standard for direct radical identification, its requirement for specialized instrumentation limits its routine in vivo deployment [25].

Ultimately, method selection must be guided by the specific biological hypothesis. To navigate the limitations of individual assays, we recommend that future preclinical studies in ovarian cancer adhere to rigorous validation practices. These include specifying the targeted ROS compartment, utilizing appropriate pharmacologic or genetic controls (e.g., NOX inhibitors, catalase, specific scavengers like MitoTEMPO, or ferroptosis inhibitors), and corroborating intensity-based fluorescence data with at least one chemically distinct, orthogonal assay (such as downstream macromolecular footprints). By doing so, ROS can be accurately evaluated as a mechanistic driver rather than a simplistic binary variable.

### 3.4. Reconciling the Dual Role of ROS in Ovarian Cancer

Apparent contradictions in the ovarian cancer literature—indicating that ROS either promote tumor progression or enforce tumor control—can be resolved by situating the findings within a multidimensional redox framework defined by thresholds (hormesis), chemical species, subcellular localization and timing, and the contextual state of the tumor and microenvironmental buffering.

#### 3.4.1. Thresholds and Hormesis

At low to moderate levels, ROS operate within a pro-adaptive window in which signaling is favored over damage [47]. In this regime, relatively stable and diffusible oxidants, especially H_2_O_2_, reversibly oxidize redox-active cysteines on phosphatases and transcriptional regulators to sustain PI3K/AKT, NF-κB, and hypoxic programs [48]. Epithelial ovarian cancers often upregulate glutathione- and thioredoxin-based buffering, which expands this adaptive window and supports proliferation, EMT, and drug tolerance. Consistent with this, increases in ROS that remain coupled to intact buffering can drive progression via inflammatory/metastatic axes. This is exemplified by mitochondrial antioxidant collapse (SOD2 knockdown), which elevates O_2_•^−^ and accelerates xenograft growth [11], or by RUNX1-IT1-mediated GPX1 repression, which raises ROS and activates NF-κB to enhance peritoneal metastasis [29]. Likewise, metabolic erosion of NADPH supply (e.g., CPT2 downregulation) elevates ROS and reinforces NF-κB signaling to confer growth and metastatic advantages [30].

#### 3.4.2. Species Specificity and Chemistry

When oxidative flux overwhelms buffering, biology crosses from adaptation to injury. Highly reactive species such as •OH (generated via Fenton chemistry) and excessive mitochondrial O_2_•^−^ are indiscriminate oxidants that impose irreversible lesions on DNA, lipids, and proteins to precipitate apoptosis, pyroptosis, or ferroptosis. The results from multiple in vivo studies illustrate this transition. For example, ACLY inhibition activates AMPK with a concurrent ROS surge to suppress PI3K–AKT signaling and curb growth while resensitizing tumor cells to cisplatin [14]. CBL0137 elevates ROS and engages a BAX–caspase-3–GSDME cascade consistent with pyroptotic execution [21]. Artesunate, an endoperoxide that amplifies iron-dependent oxidative stress, reduces tumor burden with ferroptosis-relevant features [13]. In these settings, ROS do not simply rise; rather, they breach the effective antioxidant capacity to force engagement of death programs. Importantly, the species also dictates downstream logic: H_2_O_2_ can maintain signaling in the adaptive zone, whereas lipid peroxyl radicals drive ferroptotic commitment once glutathione/GPX4 defenses are compromised.

#### 3.4.3. Subcellular and Temporal Context

ROS localization and exposure kinetics shape fate decisions. Mitochondrial ROS intimately couple to membrane depolarization and intrinsic apoptosis, while cytosolic or NOX-derived ROS frequently feed into inflammatory and DDR circuits that support survival under chronic stress. Acute, high-amplitude bursts of ROS are more likely to exceed buffering and trigger death, whereas chronic, moderate increases promote compensatory upregulation of antioxidants and prosurvival pathways. This understanding helps reconcile why the same nominal “ROS increase” can either potentiate metastasis (e.g., by sustaining NF-κB activation downstream of RUNX1–IT1) or drive tumor regression when delivered as an acute, overwhelming insult (e.g., redox-cycling or endoperoxide-based therapeutics) [13,29].

#### 3.4.4. Tumor and Microenvironmental Buffering Circuits

The outcome of ROS modulation reflects not only tumor-intrinsic defenses (GPX1, SOD2, SLC7A11/GPX4, FAO–NADPH) but also microenvironmental conditions (pH, oxygenation, myeloid polarization) and ROS-sensing networks (e.g., ATR–Chk1, CAMK2G–ITPKB). For example, neutralizing acidity with NaHCO_3_ reprograms cGMP-PKG signaling, enhances ROS scavenging, and increases M1 macrophage infiltration, thereby augmenting olaparib efficacy despite lowering tumor ROS readouts [19]. Conversely, circuits that convert ROS into survival signals (e.g., DUOXA1–ATR–Chk1 or CAMK2G–ITPKB) can maintain platinum resistance, and dismantling these circuits restores chemosensitivity even when total ROS is not the nominal target [27,28]. Thus, “increase versus decrease” is an insufficient descriptor; the decisive variables are which species are generated at what location and for how long, and whether redox-buffering and ROS-sensing pathways transduce the signal into survival or death.

#### 3.4.5. Therapeutic Implications

This conceptual synthesis offers critical therapeutic implications for overcoming the “ROS paradox” in ovarian cancer. Fundamentally, ROS must be interpreted along a dynamic continuum, where the inflection point between cellular adaptation and cytotoxicity is strictly dictated by the tumor’s redox-buffering capacity and specific pathway engagement [49]. Methodologically, future therapeutic interventions must be rationally designed with the precise redox species and subcellular compartment in mind. This entails pairing ROS-modulating agents with synergistic modifiers that either definitively push the tumor past the oxidative death threshold or, alternatively, selectively dismantle pro-survival ROS signaling networks in metastatic and DDR-dependent states. Ultimately, mapping the diverse in vivo phenotypes reported in the reviewed studies onto these multidimensional axes provides a coherent framework for translating complex redox biology into precise therapeutic strategies [50].

### 3.5. Limitations of Preclinical Models and the Influence of Model Selection on ROS Outcomes

Interpretation of in vivo ROS biology fundamentally depends on the constraints of the chosen model system, including those of its immunological competence, anatomical site, and readout methodology. Immunodeficient human CDX/PDX models elegantly isolate tumor-intrinsic redox vulnerabilities, such as mitochondrial antioxidant collapse or metabolic rewiring leading to altered growth profiles [11]. However, they inherently obscure ROS–immune crosstalk. Conversely, immunocompetent syngeneic models capture this bidirectional regulation. For instance, neutralizing TME acidity in ID8 mice was found to reduce tumor ROS signals but improved olaparib efficacy via M1 macrophage enrichment [19]. Thus, the same ROS-targeting agent may act as a direct cytotoxin in immunodeficient mice but a TME immunomodulator in immunocompetent hosts. Subcutaneous implantation yields models that lack the unique fluid dynamics, hypoxia, and iron gradients characteristic of the peritoneal cavity. Consequently, while subcutaneous models are instrumental for mapping targeted ROS amplification to intrinsic cell death pathways, they fail to fully recapitulate the peritoneal microenvironment [14,21]. In contrast, orthotopic and peritoneal models better reflect disease dissemination, such as by showing that lncRNA-driven GPX1 repression drives NF-κB-mediated peritoneal metastasis [29]. In vivo ROS probes are highly sensitive to levels of tissue perfusion, esterase activity, and phagocytosis, which can vary significantly across models and implantation sites. Apparent differences in “total ROS” may therefore partly reflect probe pharmacokinetics rather than a bona fide oxidative flux. Similarly, ferroptosis-relevant lipid peroxidation heavily depends on dietary iron and stromal composition, yet these variables are often blunted in immunodeficient models. To navigate these limitations, investigators must align model selection with their primary biological question, such as by favoring xenografts/PDXs for examining tumor-intrinsic cytotoxicity and immunocompetent orthotopic models for exploring ROS–TME interactions. Furthermore, triangulating findings across multiple platforms will help researchers critically reconcile discrepant outcomes and accelerate the translational fidelity of ROS-targeted therapies in ovarian cancer.

### 3.6. Statistical Limitations and Translational Reproducibility

A pervasive limitation across the in vivo literature synthesized in this review is the reliance on relatively small animal cohorts, typically ranging from *n* = 5 to 8 per experimental arm. While such sample sizes align with ethical imperatives regarding animal welfare and logistical constraints, they inherently reduce statistical power and increase the likelihood of overstating true biological signals. This statistical vulnerability is particularly amplified in redox biology due to the high intrinsic variability of in vivo ROS measurements, which are frequently confounded by transient oxidant half-lives and heterogeneous microenvironmental buffering. Consequently, nominally significant tumor suppression observed in underpowered, genetically homogeneous murine models often fails to capture the profound inter- and intra-tumoral heterogeneity characteristic of human epithelial ovarian cancers, ultimately contributing to high rates of clinical attrition.

To bridge this translational gap and mitigate inherent statistical risks, future preclinical studies must adopt more rigorous methodological frameworks. Primarily, investigators should implement a priori power calculations based on minimally important clinical differences, ensuring that cohorts are adequately powered to detect robust biological effects. This prospective design must be strictly coupled with true random allocation and blinded outcome assessments to minimize unconscious bias. Furthermore, applying robust statistical modeling is essential; researchers must treat the individual animal as the fundamental unit of inference to avoid the pitfalls of pseudoreplication. Exploiting repeated-measures designs with baseline adjustments for longitudinal tumor analyses, alongside the explicit reporting of effect sizes and 95% confidence intervals rather than relying exclusively on isolated *p*-values, will provide a more transparent and reproducible assessment of therapeutic efficacy.

Beyond refined statistical practices, improving translational reliability necessitates orthogonal pharmacodynamic validation and rigorous model triangulation. Because standard fluorescent ROS readouts can be noisy or compartmentally restricted, tumor-growth outcomes should be consistently paired with mechanistically aligned biomarkers. Validating stable oxidative-damage footprints—such as 8-OHdG, γ-H2AX, or 4-HNE adducts—or applying precise ferroptosis-rescue criteria can corroborate the presence of lethal oxidative stress without relying on a single assay [51]. Finally, mechanistic findings initially mapped in immunodeficient subcutaneous xenografts must be systematically cross-validated in orthotopic or immunocompetent systems. Benchmarking these ROS-modulating interventions against standard-of-care chemotherapies across diverse immunological and anatomical contexts will robustly test their therapeutic resilience. By elevating statistical rigor and standardizing these redox pharmacodynamics, the field can better discriminate durable therapeutic effects from context-dependent noise, thereby accelerating the successful clinical translation of ROS-targeted strategies in ovarian cancer.

### 3.7. Clinical Translation and Therapeutic Windows in Ovarian Cancer

The successful clinical translation of ROS-modulating therapies will require the identification of a well-defined therapeutic window. Epithelial ovarian cancers characteristically exhibit constitutively elevated basal oxidative stress, and profoundly rely on antioxidant defense systems—primarily the glutathione and thioredoxin networks—to maintain cellular viability [52]. This fundamental dependency establishes a critical therapeutic asymmetry: The targeted amplification of ROS or the pharmacological disruption of these antioxidant axes can selectively exceed the cytotoxicity threshold of malignant cells. Conversely, normal tissues remain largely unaffected due to their substantial reserve-buffering capacity. Importantly, this redox vulnerability functionally converges with current standard-of-care regimens: Platinum-based chemotherapeutics inherently elevate the intracellular ROS burden, whereas PARP inhibitors exacerbate unrepaired oxidative DNA lesions. Therefore, the field must seek to develop mechanism-driven combinatorial strategies. Promising modalities include coupling targeted generation of ROS with inhibition of DDR signaling cascades, dismantling the SLC7A11/GPX4 axis to precipitate extensive lipid peroxidation and ferroptosis, and modulating the acidity of the TME to optimize immune system–drug synergy [19,46].

Successfully bridging these strategies to the clinic will require multi-tiered biomarker stratification and rigorous early-phase trial execution. Because direct ROS quantification is highly labile, patient selection and pharmacodynamic monitoring must rely on stable, orthogonal readouts. These include markers of tumor-intrinsic buffering capacity, stable oxidative stress footprints, and tumor microenvironment immune profiles [25]. Operationally, early-phase trials should employ fractionated or sequence-controlled dosing—such as “redox priming” followed by DDR inhibition—to maximize synthetic lethality while permitting normal-tissue recovery. This must be coupled with predefined pharmacodynamic panels and time-locked biopsies to confirm on-target engagement. Furthermore, strict safety protocols will be required to monitor baseline oxidative toxicity and control for drug–nutrient interactions. In particular, high-dose antioxidant supplements could be used to negate therapeutic efficacy. Ultimately, achieving durable clinical translation will rest on the ability to precisely match the ROS-modulating intervention to the patient’s basal redox state and DDR/tumor microenvironment context, with continuous validation using robust, multi-marker pharmacodynamic frameworks.

## 4. Conclusions

**Figure 2:**
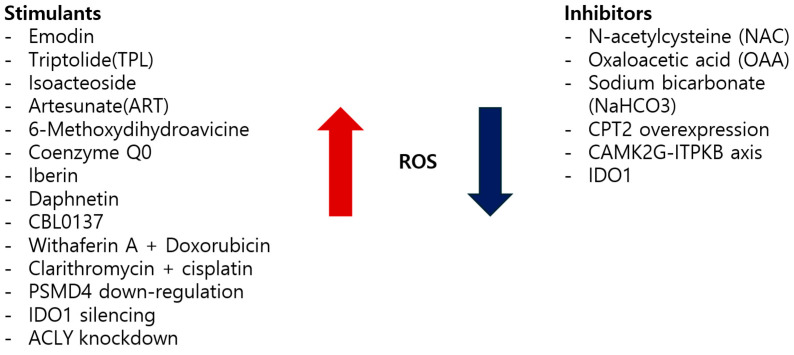
In vivo modulators of reactive oxygen species (ROS) in ovarian cancer.

Taken together, the results of the 22 in vivo studies included in this review indicate that the relationship between ROS and anticancer responses in ovarian cancer is determined less by a simple correlation with increased or decreased ROS and more by context—namely, whether ROS modulation coincides with changes in tumor burden, whether it co-occurs with oxidative stress and/or cell-death markers, and whether the antitumor phenotype is attenuated or rescued when ROS itself or downstream death/resistance circuits are experimentally manipulated. The specific in vivo modulators—including pharmacological agents and genetic interventions—that act as either ROS stimulants or inhibitors across these models are summarized in Figure 2. First, across multiple xenograft and resistance models, ROS amplification (or weakening of antioxidant buffering capacity) was consistently accompanied by apoptosis and suppression of survival signaling, and was frequently reported to restore or enhance platinum responsiveness. Moreover, ROS was positioned as an upstream signal not limited to apoptosis but also extending to other cell-death modalities, including pyroptosis (ROS–BAX–caspase-3–GSDME) and lethal autophagy (the ROS–JAK2/STAT3–Beclin1 axis). These findings support the preclinical plausibility of combination strategies such as ROS modulation plus simultaneous targeting of execution pathways (or cytoprotective autophagy). Second, in the opposite direction, the in vivo data also support axes in which elevated ROS culminates in tumor promotion, metastasis, and/or maintenance of resistance. Reduced levels of the antioxidant enzyme, SOD2, can accelerate tumor growth together with superoxide accumulation. Additionally, ROS–inflammatory signaling circuits have been linked to increased metastatic burden, as exemplified by CPT2 downregulation with ROS/NF-κB activation and RUNX1-IT1-mediated GPX1 suppression leading to ROS accumulation and NF-κB activation. Third, tumor promotion can be achieved not only through “increased” ROS, but also through enhanced redox buffering that enables ferroptosis evasion. In orthotopic models, the CEBPG–SLC7A11 axis was functionally linked to tumor maintenance based on rescue evidence, suggesting that the importance of ROS should be reframed not merely as reflecting a change in total ROS levels but as the function of an axis centered on lipid-ROS/ferroptosis susceptibility. In addition, the artesunate model, combining in vivo tumor suppression with mechanistic data involving iron and ferrostatin-1, proposes that iron-dependent oxidative damage (with ferroptosis as a plausible modality) may represent a therapeutically exploitable vulnerability.

In conclusion, ROS-targeted strategies in ovarian cancer should not be reduced to a binary decision of whether to increase or decrease ROS. Instead, they should be refined into context-dependent combination designs that integratively consider: (1) the redox-buffer systems on which tumors rely (e.g., GPX1/SLC7A11/FAO–NADPH), (2) ROS-sensing/buffering circuits and DNA damage responses (e.g., ROS-sensing circuits), and (3) cell-death mechanisms (apoptosis/pyroptosis/lethal autophagy/ferroptosis).

## Data Availability

No new data were created or analyzed in this study. Data sharing is not applicable to this article.
